# Emerging role of lncRNAs in drug resistance mechanisms in head and neck squamous cell carcinoma

**DOI:** 10.3389/fonc.2022.965628

**Published:** 2022-08-01

**Authors:** José A. Peña-Flores, Mercedes Bermúdez, Rosalío Ramos-Payán, Carlos E. Villegas-Mercado, Uriel Soto-Barreras, Daniela Muela-Campos, Alexis Álvarez-Ramírez, Brenda Pérez-Aguirre, Ana D. Larrinua-Pacheco, César López-Camarillo, Jorge A. López-Gutiérrez, Julio Garnica-Palazuelos, Marvin E. Estrada-Macías, Juan L. Cota-Quintero, Andrés A. Barraza-Gómez

**Affiliations:** ^1^ Faculty of Odontology, Autonomous University of Chihuahua, Chihuahua, Mexico; ^2^ Faculty of Biological and Chemical Sciences, Autonomous University of Sinaloa, Culiacán, Mexico; ^3^ Autonomous University of Mexico City, Mexico City, Mexico; ^4^ Faculty of Biology, Autonomous University of Sinaloa, Culiacán, Mexico; ^5^ Faculty of Odontology , Autonomous University of Sinaloa, Culiacán, Mexico

**Keywords:** lncRNA, autophagy, cancer, EMT, stemness, HNSCC (head and neck squamous cell carcinoma), drug resistance, chemoresistance

## Abstract

Head and neck squamous cell carcinoma (HNSCC) originates in the squamous cell lining the mucosal surfaces of the head and neck region, including the oral cavity, nasopharynx, tonsils, oropharynx, larynx, and hypopharynx. The heterogeneity, anatomical, and functional characteristics of the patient make the HNSCC a complex and difficult-to-treat disease, leading to a poor survival rate and a decreased quality of life due to the loss of important physiologic functions and aggressive surgical injury. Alteration of driver-oncogenic and tumor-suppressing lncRNAs has recently been recently in HNSCC to obtain possible biomarkers for diagnostic, prognostic, and therapeutic approaches. This review provides current knowledge about the implication of lncRNAs in drug resistance mechanisms in HNSCC. Chemotherapy resistance is a major therapeutic challenge in HNSCC in which lncRNAs are implicated. Lately, it has been shown that lncRNAs involved in autophagy induced by chemotherapy and epithelial–mesenchymal transition (EMT) can act as mechanisms of resistance to anticancer drugs. Conversely, lncRNAs involved in mesenchymal–epithelial transition (MET) are related to chemosensitivity and inhibition of invasiveness of drug-resistant cells. In this regard, long non-coding RNAs (lncRNAs) play a pivotal role in both processes and are important for cancer detection, progression, diagnosis, therapy response, and prognostic values. As the involvement of more lncRNAs is elucidated in chemoresistance mechanisms, an improvement in diagnostic and prognostic tools could promote an advance in targeted and specific therapies in precision oncology.

## Introduction

Cancer is a group of multifactorial diseases with an estimated 9.9 million deaths globally in 2020 (1). HNSCC is the sixth most common cancer in the world, accounting for more than 850,000 cases and 400,000 deaths every year (1). HNSCC originates in the squamous cell lining the mucosal surfaces of the head and neck region, involving the oral cavity, nasopharynx, tonsils, oropharynx, larynx, and hypopharynx (2, 3). The main risk factors related to them are smoking, alcohol consumption, betel nuts, smokeless tobacco, and viral infections, including Epstein–Barr and human papillomavirus (4). Nowadays, the treatment for advanced HNSCC includes chemotherapeutic agents, radiotherapy, and surgical resection, leading to mutilation of essential tissues that affect functions such as breathing, feeding, and speaking, thus decreasing the quality of life of patients (5). The heterogeneous nature of HNSCC leads to a poor 5-year overall survival rate due to its local invasion, chemoresistance, metastasis, and late diagnosis (2, 6).

Chemotherapy has been widely used in recent decades for cancer treatment. The combination of platinum-based 5-fluorouracil (5-FU) and DNA synthesis inhibitor cisplatin (CDDP) is still the main regimen for HNSCC (5). However, combinations like paclitaxel (PTX), carboplatin (CDBCA), and cetuximab have been proposed, with unpredictable results (7, 8). Recently, immune checkpoint blockade (ICB) treatment is gaining importance as an immunologic approach for cancer control. HNSCC has a high tumor mutational burden and a relatively high expression of programmed cell death-1-ligand 1 (PD-L1), making it eligible for ICB (9, 10). Nevertheless, drug resistance (DR) is still a key factor for HNSCC progression and poor prognosis (11). The detailed mechanisms of DR are not fully understood, but recent studies suggest that autophagy (12–14), epithelial–mesenchymal transition (EMT) (13, 15, 16), and cancer cell stemness (17–19) play a pivotal role in this major problem. Other mechanisms implied in DR are inactivation of the drug, multi-drug resistance, apoptosis suppression, alterations in the drug metabolism, epigenetic changes, changes in the drug targets, enhanced DNA-repair, and target gene amplification (20). Besides, there are also biological determinants of drug resistance such as tumor heterogeneity, physical barriers, immune system and tumor microenvironment, undruggable cancer drivers, and selective therapeutic pressure that induces changes in the tumor and its ecosystem, modifying the response of the cells to different drugs (21).

Recent studies indicate that non-coding RNAs (ncRNAs) comprise 98% of the total transcribed RNAs in the human genome, and although at first they were classified as “junk” transcriptional products, nowadays they play crucial roles in many biological processes modulating gene expression (22, 23). ncRNAs dysregulation contributes to an increasing number of human diseases, including cancer (2). Long non-coding RNAs (lncRNAs) are a class of functional RNA composed of at least 200 nucleotides (24). LncRNAs have a high transcriptional rate as they are involved in gene regulation at the transcriptional level in the nucleus and posttranscriptional level in the cytoplasm (25, 26). Moreover, lncRNAs are implicated in various cancer progression mechanisms, including proliferation, differentiation, autophagy, EMT, invasion, and metastasis (27–29). Increasing evidence suggests that lncRNAs are implicated in DR in different types of cancer, including HNSCC (30–33). In this regard, this review provides current knowledge about lncRNAs and their implication in DR through known processes in HNSCC, emphasizing in autophagy, EMT, and cancer cell stemness mechanisms. A systematic search was performed in PubMed, Web of Science, Google Scholar, Cochrane Library, and Embase from 2017 to May 2022 for articles matching the following criteria: (long non-coding RNA (lncRNA) and (head and neck squamous cell carcinoma (HNSCC), or oral cancer, or oral squamous cell carcinoma (OSCC), or buccal cancer, or lip cancer or tongue cancer (TSCC) or pharyngeal carcinoma, or nasopharyngeal carcinoma (NPC) or laryngeal squamous cell carcinoma (LSCC)), and (chemoresistance or drug resistance or cisplatin resistance or CDDP resistance), and (autophagy or epithelial-mesenchymal transition or EMT or stemness or cancer stem cells (CSCs)). The titles and abstracts were screened and acquired relevant full-text manuscripts were for further analysis.

## Long non-coding RNAS

### Biogenesis, classification, and function

Approximately 93% of the human genome can be transcribed into RNAs, but only 2% of these transcripts are translated into proteins; the remaining 98% are ncRNAs (24). LncRNAs, also referred to as competing endogenous RNAs (ceRNAs) (34), include different kinds of RNA polymerase II (Pol II)-transcribed molecules, mostly 5’-capped, polyadenylated, and spliced (35), and studies suggest there could be more than 10,000 lncRNA transcripts in humans (36, 37). LncRNAs are engaged in multiple functions, including the modulation of crucial functions of other ncRNAs such as micro-RNAs (miRNAs), small nucleolar RNAs (snoRNAs), etc. (38).

**Figure 1:**
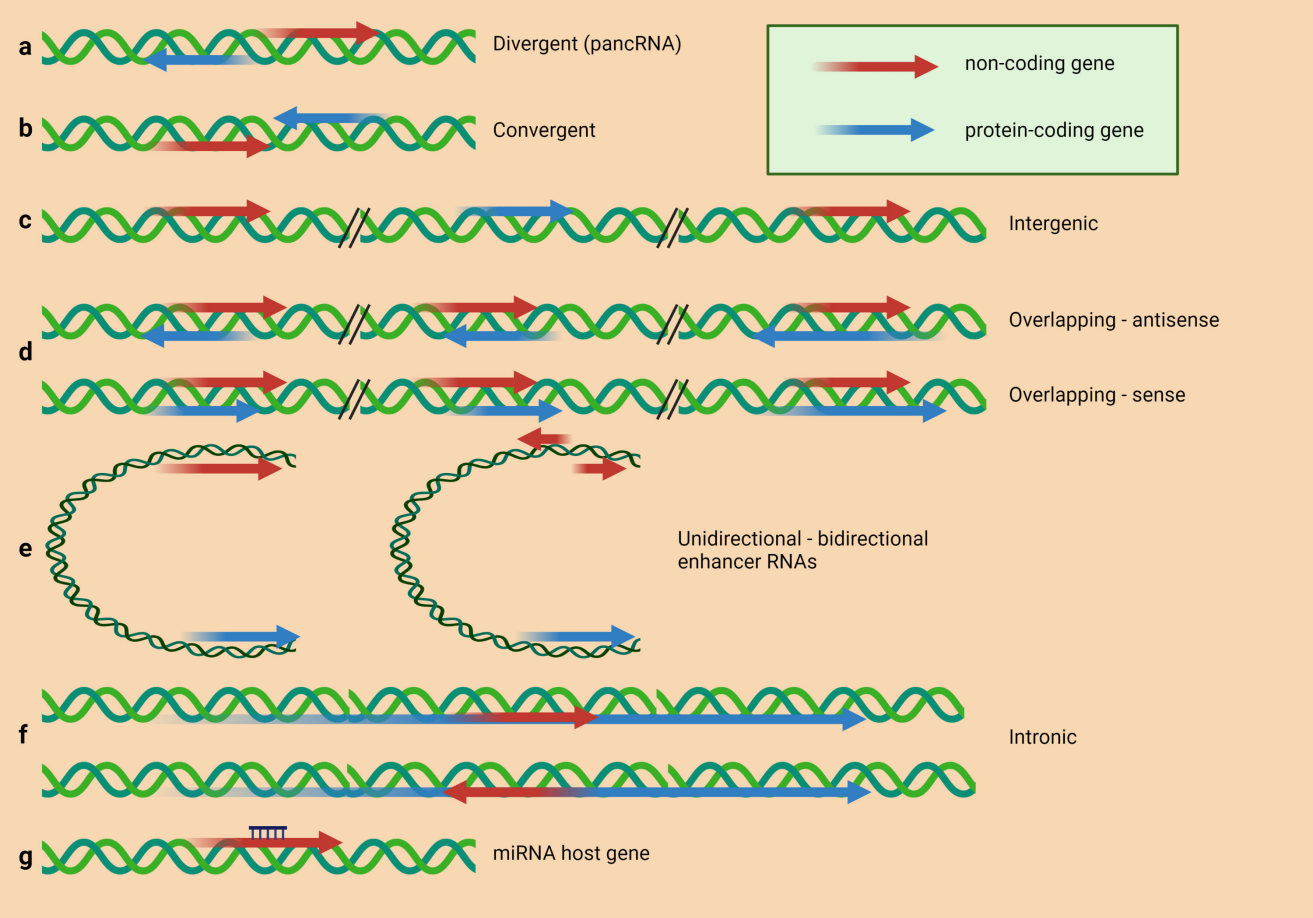
LncRNAs classification based on their structural origin. According to Schmitz et al. (35), lncRNAs can be classified in **(A)** divergently transcribed lncRNA originating from the same promoter region as the adjacent protein-coding gene, but from the opposite strand; **(B)** genes encoded on opposite strands, facing each other and convergently transcribed; **(C)** intergenic lncRNA (lincRNA) located distant from other genes; **(D)** lncRNAs overlapping with other genes on the same or opposite strand; **(E)** enhancer RNAs expressed as uni- or bidirectional transcripts; **(F)** lncRNA transcribed from an intron of another gene; **(G)** lncRNA hosting miRNA.

Advances in RNA sequencing and other techniques have allowed the discovery of an increasing number of lncRNA classes based on diverse parameters such as transcript length, mRNA resemblance, biogenesis, and unique regulatory mechanisms, among others (39, 40). According to Schmitz et al., one of the most used categorizations is related to the position of the lncRNAs in the genome relative to protein-coding genes (**Figure 1**) (35). The lncRNA can be divergent (pancRNA) when the lncRNA and neighboring protein-coding gene are transcribed in opposite strands (41), convergent when the lncRNA and protein-coding gene neighbor are transcribed to the same point (42), intergenic when a lncRNA sequence belongs to two genes as a distinct unit (43), overlapping when a protein-coding gene is included in the intron of the same lncRNA in sense or antisense orientation (44), enhancer RNAs expressed as uni- or bidirectional transcripts (45), intronic when the sequence of the lncRNA belongs to the intron of a protein-coding gene (46). Some lncRNAs are generated by back splicing from introns of mRNAs or other lncRNAs and are thus circular (circRNAs) (47).

An increasing number of lncRNAs have been associated with both important biological functions and pathological conditions such as diabetes, neurodegenerative diseases, rheumatoid arthritis, cardiovascular diseases, and cancer (48–50). Dahariya et al. suggest that activation and inhibition of gene expression are promoted by lncRNAs through diverse molecular mechanisms comprising of four basic mechanisms: signal, decoy, guide, and scaffold. In this regard, recent evidence suggests that signaling mediators like kinases, receptors, and transcription factors are strongly associated with lncRNAs *via* numerous signaling pathways, such as PI3K/AKT/mechanistic target of rapamycin (mTOR), Wnt, and the MAPK signaling pathways (51–53). On the other hand, decoy lncRNAs can diminish the availability of regulatory factors by presenting binding sites (54). For instance, Zhang et al. (55) demonstrated that LINC00160 functions as a decoy of miRNA-132 targeting PIK3R3 to mediate DR in hepatocellular carcinoma, whereas lncRNA GAS5 can also act as a molecular sponge that blocks their downstream functions by targeting RNA or proteins (56). Besides, lncRNAs can interact with ribonucleoproteins (RNPs) in the genome to guide their precise localization, such as HOTAIR, which directs PRC2 to the HOXD locus, leading to silencing genes involved in metastasis suppression (54, 57). Also, CASC9 acts as a guide for EZH2 and CREB-binding protein (CBP) to the promoter regions of target genes (58). In the case of scaffold lncRNAs, they can act as the central platform for assembling complexes, for example, by binding to RNP K and EZH2 to induce the formation of a complex to repress SOX2 (51, 54, 59). Overall, the diverse functions of lncRNAs depend on their subcellular location (60). Genome sequencing has shown that a large proportion of lncRNAs are localized in the nucleus or associated with chromatin, whereas the remaining fraction is localized in the cytoplasm (61).

**Figure 2:**
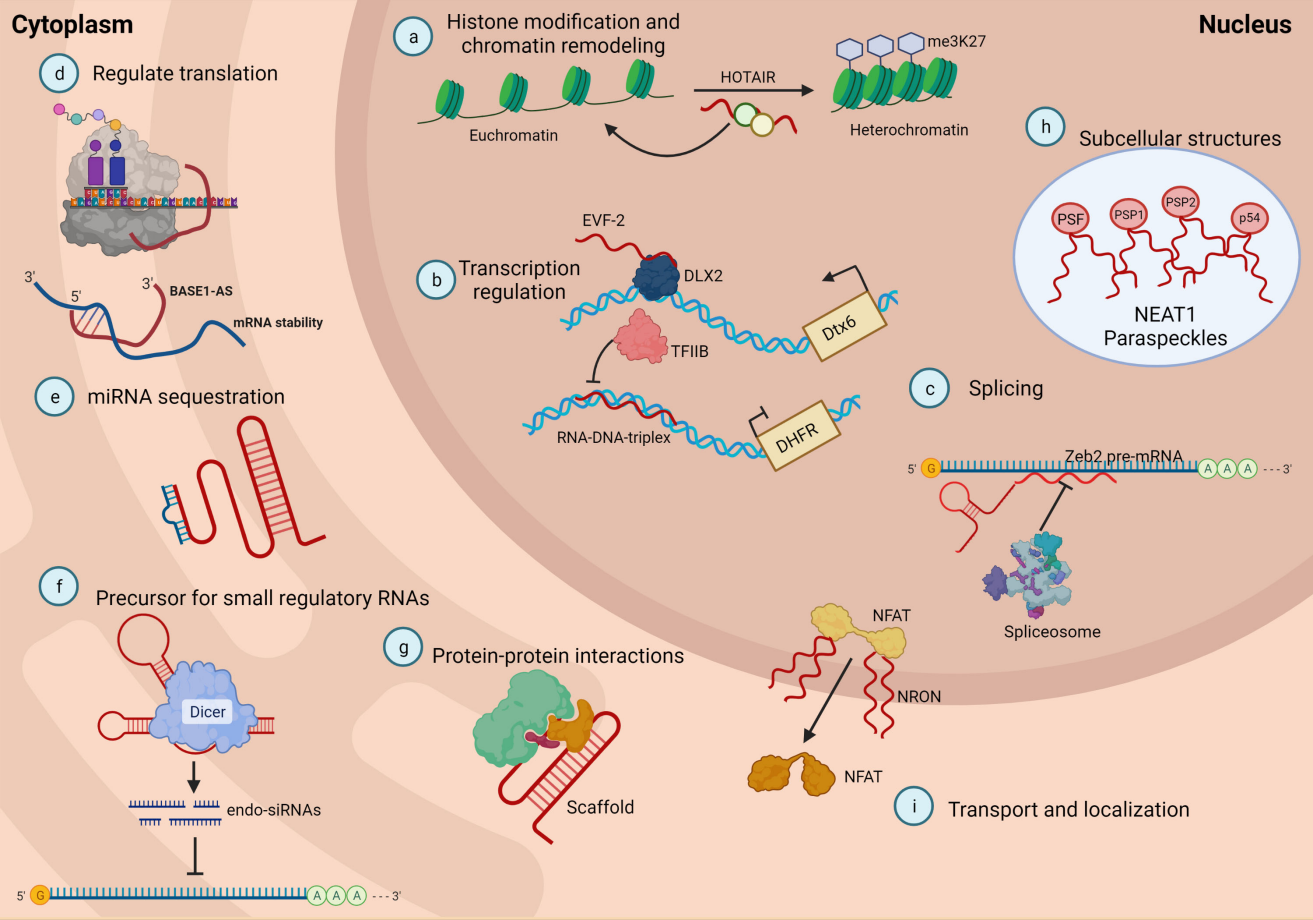
LncRNAs can be classified based on their functions. **(A)** lncRNA can guide chromatin complexes controlling between transcriptionally active euchromatin and silent heterochromatin; **(B)** the recruitment of polymerase II and transcription factors can be inhibited or facilitated by lncRNAs; **(C)** lncRNAs contribute to transcriptome complexity by regulating alternative splicing of pre-mRNAs; **(D)** lncRNAs affect the stability and translation of mRNA by base pairing with mRNA molecules; **(E)** they influence in the expression of miRNAs by binding to them and preventing their function; **(F)** lncRNAs can act as siRNAs and target other RNAs, which subsequently could result in target degradation; **(G)** lncRNAs can join multiple protein factors as flexible scaffolds to interact or cooperate on protein-protein interactions; **(H), (I)** the scaffold function is also important for protein activity and localization as well as subcellular structures. Adapted from Meng et al. (64).

Many studies have demonstrated the crosstalk of lncRNAs with many epigenetic factors to regulate gene expression and modulate nuclear structure by facilitating the architecture of nuclear speckles, paraspeckles, and interchromatin granules (36, 62). Some lncRNAs play a role as regulators to initiate, elongate or terminate actions of transcription factors (38). Other types of lncRNAs act as decoys by binding to transcription factors or proteins and deviating from protein factors in their action on target DNA (37). They also act as sponges or molecular sinks for miRNAs, mediating changes in gene expression by acting on transcription factors, cell receptors, growth factors, and splicing regulators (63). The main functions of lncRNAs are depicted in **Figure 2**.

### Role of lncRNAs in cancer

Recently, an increasing number of studies of high RNA-sequencing have provided resources for the identification of many lncRNAs that are dysregulated in solid tumors (65–67). LncRNAs are often found as regulators in tumorigenesis, progression, and metastasis of cancer by modulating signaling cascades at the epigenetic, transcriptional, posttranscriptional, translational, or posttranslational levels (65). Cancer-controlling lncRNAs are categorized as proto-oncogenic or tumor suppressors based on their function, being the tumorigenic lncRNAs expressed in tumors as cancer drivers that activate the cell cycle, promote proliferation, and/or exert anti-apoptosis effects (65, 68). Moreover, cancer-progressing lncRNAs have been related to EMT, cell migration, and cell invasion (65, 68). Approximately 100 lncRNAs have been identified recently as regulators of the development and progression of multiple cancer types, including prostate (69–72), breast (73–75), lung (76–79), colorectal (80–82), liver (83–85), and leukemia (86–88), among others.

On the other hand, many lncRNAs have been documented as tumor suppressors and they are generally downregulated in tumor biopsies compared with their normal counterparts (65). When these tumor-suppressing lncRNAs are downregulated or suppressed, they can lead to increased proliferation and tumor growth (65). Although many tumor-suppressing lncRNAs are under investigation, the most documented are the growth arrest-specific transcript 5 (GAS5) (89), the maternally expressed gene 3 (MEG3) (90), and the NF-kB interacting lncRNA (NKILA) (91).

### LncRNAs in HNSCC

HNSCC comprises a group of cancers that originate in the squamous-cell layer of the mucosa lining in the head and neck region. The heterogeneity, anatomical, and functional features make the HNSCC a complex and difficult-to-treat disease, leading patients to a poor survival rate and a decreased life quality due to the loss of important physiologic functions and aggressive surgical mutilation (2, 24, 25). Alteration of driver-oncogenic and tumor-suppressing lncRNAs has been recently studied in HNSCC to obtain possible biomarkers for diagnostic, prognostic, and therapeutic approaches (2, 24, 25). **Table 1** summarizes oncogenic and tumor suppressor lncRNAs commonly found in HNSCC.

**Table 1:** Overview of proto-oncogene and tumor-suppressor lncRNAs involved in head and neck cancers.

LNCRNA	TARGET	FUNCTION	REFERENCE
**OSCC**			
**CASC9**	AKT/mTOR pathway	Enhances cell proliferation and suppresses autophagy-mediated cell apoptosis.	(92)
**GALAT1**	miRNA-149	It Promotes proliferation and migration, and inhibits apoptosis and autophagy.	(93)
**LINC01207**	miR-1301-3p	It Promotes cell proliferation, migration, and inhibits apoptosis and autophagy.	(94)
**HOTAIR**	MAP1L3B, Beclin1, ATG3, and ATG7	Its silencing promoted proliferation, migration, and invasion.	(95)
**LINC00958**	miR-4306	Its silencing suppressed cell proliferation, induced cell death, and reduced autophagy.	(96)
**PTCSC3**	ND	Its overexpression caused a significant decrease in invasion.	(29)
**UCA1**	miR-184	Accelerates proliferation, increases cisplatin (CDDP) chemoresistance, and restrains apoptosis.	(97)
**HOXA11-AS**	miR214-3p/PIM1 axis	Promotes proliferation and inhibits cisplatin-induced cytotoxicity.	(98)
**XIST**	miR-27b-3p	Promotes proliferation, CDDP resistance, and inhibits apoptosis.	(99)
**MALAT1**	PI3K/AKT/m-TOR pathway	Induces EMT and CDDP resistance.	(100)
**ANRIL**	ND	Increases anti-apoptotic protein Bcl-2 expression.	(101)
**OIP5-AS1**	miR-27b-3p	Its knockdown enhanced CDDP sensitivity.	(102)
**KCNQ1OT1**	miR-124-3p miR-211-5p	Its knockdown inhibited survival rate, proliferation, migration, invasion, and EMT. Facilitates tumor growth and chemoresistance.	(28) (103)
**SNHG26**	AKT/m-TOR pathway	Promotes proliferation, EMT, migration, invasion, and CDDP resistance.	(104)
**CYTOR**	miR-1252-5p and miR-3148	Promotes EMT and chemoresistance	(15)
**LHFLP3-AS1**	miR-194-5p	Its knockdown suppresses proliferation, migration, and invasion.	(105)
**CEBPA-DT**	ND	Its downregulation enhances cisplatin sensitivity.	(106)
**MPRL**	Pre-miR-483	High expression is associated with chemosensitivity and a better prognosis.	(107)
**PVT1**	miR-194-5p	Correlated with worse overall survival and CDDP resistance.	(108)
**HEIH**	miR-3619-5p	Promotes CDDP resistance.	(109)
**CILA1**	ND	Promotes EMT, invasiveness, and chemoresistance.	(110)
**APCDD1L-AS1**	miR-1224-5p/NSD2 axis	Confers resistance to 5-FU.	(111)
**TUG1**	miR-133-b and CXCR4	Its downregulation impeded cisplatin resistance.	(112)
**LINC00953**	ABCB5	Its downregulation inhibited CSC hallmarks.	(17)
**NPC**			
**MEG3**	miR-21	Promotes autophagy and apoptosis.	(113)
**CASC19**	AMPK/m-TOR pathway and PARP1 pathway	Contributes to radioresistance and promotes apoptosis.	(114)
**ZFAS1**	miR-100-3p	Promotes cell proliferation, migration, and tumor growth.	(115)
**HOXA11-AS1**	miR-98/PBX3 axis miR-454-3p	Enhances CDDP resistance. Promotes cell apoptosis and CDDP sensitivity.	(116) (117)
**KCNQ1OT1**	miR-454/USP47 axis	Enhances CDDP resistance.	(118)
**TINCR**	INCR-ACLY-PADI1-MAPK-MMP2/9 axis	Acts as a driver of progression and chemoresistance.	(119)
**AFAP1-AS1**	miR-320a	Its silencing promoted chemoresistance.	(120)
**MIAT**	HMB1	It correlates with poor clinical outcome.	(121)
**NEAT1**	Let-7p-5p	Its inhibition represses CDDP resistance.	(122)
**LINC00346**	miR-342-5p	Its over-expression promotes CDDP resistance.	(123)
**MAGI2-AS3**	miR-218-5p/GDPD5/SEC61A1 axis	Promotes cell proliferation, migration, and EMT.	(124)
**n375709**	ND	Its inhibition increased paclitaxel sensitivity.	(125)
**NEAT1**	miR-129/Bcl-2 axis	Its depletion enhances SAHA-induced apoptosis.	(126)
**CCAT1**	miR-181a/CPEB2 axis	Enhances paclitaxel resistance.	(127)
**MRVI1-AS1**	Hippo-TAZ pathway	Increases paclitaxel chemosensitivity.	(128)
**DLEU1**	miR-381-3p	Promotes CDDP resistance.	(129)
**LSCC**			
**GAS5**	miR-26a-5p	Activates autophagy and induces apoptosis.	(130)
**H19**	miR-107	Inhibits autophagy and drug resistance.	(131)
**MALAT1**	ND	Enhances chemoresistance and poorer 5-year survival.	(132)
**FOXD2-AS1**	STAT3 and PRMT5	Predicts poor prognosis, maintains cancer stemness and promotes chemotherapeutic resistance.	(18)
**FGD5-AS1**	miR-497-5p/SEPT2 axis	Its overexpression increases CDDP resistance.	(133)
**HOXA11-AS1**	miR-518a/SPATS2L axis	Enhances CDDP resistance.	(134)
**LINC-PINT**	miR-425-5p	Its downregulation increases cancer stemness and chemoresistance to cisplatin.	(19)
**BANCR**	ND	Its downregulation reverses CDDP resistance.	(30)
**HNSCC**			
**LINC00460**	miR-206	Facilitates apoptosis and autophagy.	(33)
**PVT1**	miR-124-3p	Decreases sensitivity to cetuximab.	(135)
**LINC00461**	miR-195	Promotes EMT and chemoresistance.	(16)
**Lnc-POP-1**	VN1R5	Its upregulation promotes DNA repair.	(32)
**LINC00958**	ND	Facilitates cancer development and resistance to chemo- and radiotherapy.	(31)

Several lncRNAs have been assessed for their involvement at different stages in HNSCC. The metastasis-associated lung adenocarcinoma transcript 1 (MALAT1) is localized in the nuclear speckle periphery and it is widely studied in cancer development and progression (136). This lncRNA has been strongly associated with squamous cell carcinomas (SCC), including oral SCC (100) and laryngeal SCC (132), demonstrating poorer 5-year survival when highly expressed in tumor tissues.

A novel identified homeobox A11 antisense lncRNA (HOXA11-AS) has also been categorized as a facilitator in the process of malignant tumor proliferation and metastasis (137). Wang et al. demonstrated the proliferation of OSCC cells when HOXA11-AS was upregulated, whereas its downregulation increased apoptosis and caspase 3 activity in CDDP-resistant OSCC cells (98). Moreover, HOXA11-AS knockdown inhibited viability, migration, and invasion in LSCC and enhanced cisplatin sensitivity, thus promoting cell apoptosis in NPC tumor tissues (116, 117, 134).

In a tongue SCC study, the upregulation of the lncRNA KCNQ1 opposite strand/antisense transcript 1 (KCNQ1OT1) demonstrated a strong correlation with the survival rate, proliferation, migration, invasion, and EMT of tongue cancer cells (28). Another study by Zhang et al. found that KCNQ1OT1 facilitated tumor growth and chemoresistance by acting as a modulating ceRNA of miR-211-5p (103). Also, in NPC cell lines, the knockdown of KCNQ1OT1 promoted chemosensitivity and decreased cell proliferation, migration, and invasion by interfering with the miR-454/USP47 axis (118).

Some lncRNAs act as tumor suppressors when upregulated. In this regard, MEG3 downregulation is associated with poor survival of most cancer patients (138) since its upregulation enables the expression of tumor suppressor genes p53 and Rb, induces inhibition of angiogenesis-related factors, and can sponge miRNAs (138, 139). In HNSCC, Lin et al. demonstrated that MEG3 expression was downregulated in NPC cells, inhibiting autophagy and apoptosis ability by acting as a ceRNA to miR-21 (113). Another previously identified tumor-suppressing lncRNA related to HNSCC is the nuclear paraspeckle assembly transcript 1 (NEAT1), strongly associated with suppressing cisplatin resistance by modulating several signaling pathways like the Ras-MAPK and the miR-129/Bcl-2 axis in NPC cells (122, 126). Other lncRNAs identified as tumor suppressors in HNSCC are LINC00460 (33), GAS5 (130), MRVI1-AS1 (128), and MPRL (107). Further identification of these transcripts remains to be elucidated.

Several efforts have been made to identify valuable prognostic lncRNA signatures in different head and neck cancers. Jian et al. associated eight different lncRNAs with OSCC/OPSCC (oropharyngeal squamous cell carcinoma) prognosis, indicating a significantly lower overall survival in the high-risk group (13). Moreover, 493 HNSCC patients were screened for 363 prognostic-related lncRNAs, finding 17 lncRNAs related to the progression and prognosis of HNSCC. These differentially expressed genes (DEGs) between high- and low-risk groups are mainly enriched in immune-related pathways and regulated by a prognostic-lncRNA-directed ceRNA network (12). In a study by Li et al., 501 HNSCC cases were obtained from the National Cancer Institute GDC Data Portal and analyzed by gene set enrichment analysis (GSEA) and gene ontology (GO) functional annotation, proving that the autophagy-related lncRNA signature (LINC00958, PSMA3−AS1 UBAC2−AS1, AC008115.3, AL139 9158.2, AC136475.2, AL160006.1, AL3 57033.4, AC007991.2, AC104083.1, A L139287.1, and AL450992.2) could be considered to predict the prognosis of patients with HNSCC (140).

## LncRNAs in drug resistance mechanisms

**Figure 3:**
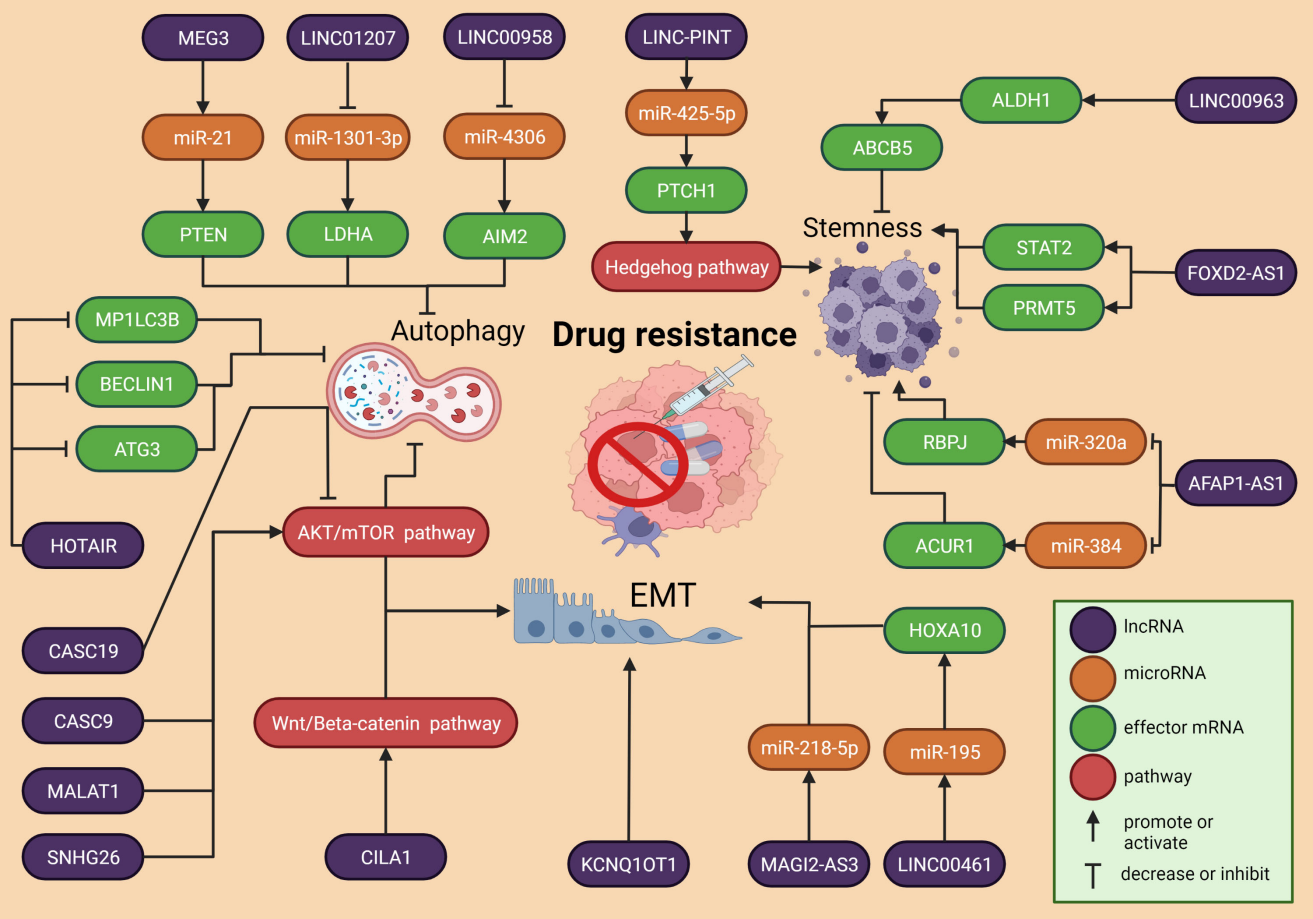
Overview of the molecular mechanisms of lncRNA in HSCC drug resistance. Three of the most important cellular processes involved with drug resistance are autophagy, EMT and stemness. All of them are ruled by regulatory axes that comprise the interaction between lncRNAs, microRNAs and expression of genes.

Three of the most important mechanisms of DR in HNSCC, autophagy, EMT, and stemness, are regulated by multiple lncRNAs (**Figure 3**). Chemotherapy remains a very common treatment option for cancer patients, although it has been established that DR is responsible for around 90% of deaths in cancer patients receiving chemotherapeutics or targeted drugs (141). After the drug is administered, the therapeutic agents pass through a phase of active intracellular metabolism along with the degradation by the liver and other metabolic organs and tissues (142). Furthermore, the dysregulation of enzymes and other proteins responsible for cellular metabolism offers additional challenges that reduce the effectiveness of anti-tumor drugs (143).

**Figure 4:**
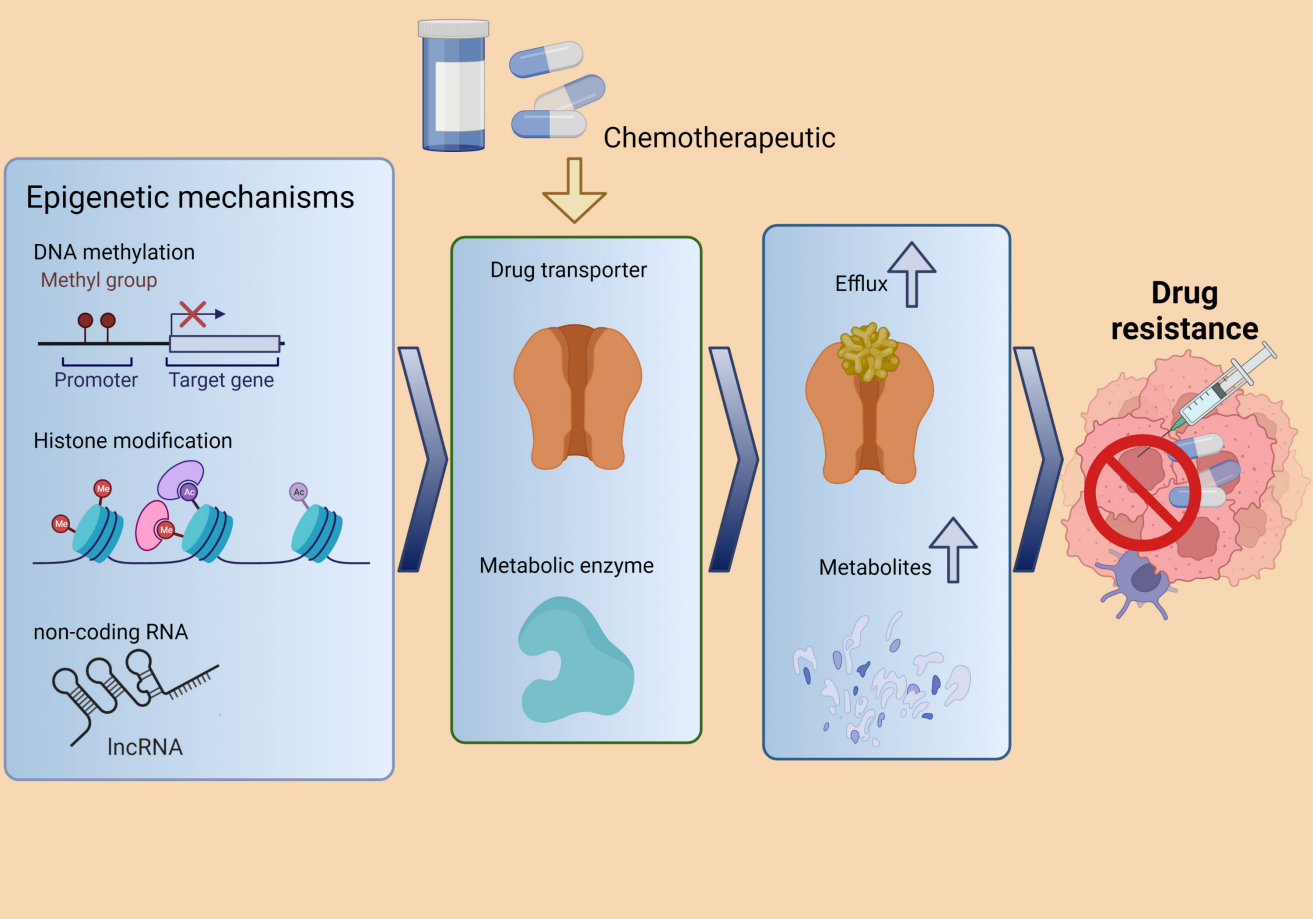
The epigenetic processes involved in cancer chemoresistance includes DNA methylation, histone acetylation, and lncRNA interaction. These processes regulate drug transporters and metabolic enzymes, promoting drug resistance. Adapted from Zhou et al. (148).

Three main phases of drug metabolism and disposition have been observed: phases I and II concerning drug metabolism and phase III concerning drug disposition (144). In phase I, enzymes are mostly cytochrome P450 (CYPs) and are involved in the oxidation, reduction, and/or hydrolysis processes that activate or inactivate the agent (145). During phase II, metabolic reactions are carried out by transferases whose primary mission is to deactivate pharmacologically active drugs, facilitating their elimination by making them more soluble in water (146). Finally, phase III consists of drug transporters active in the absorption, distribution, and elimination of drugs (147). Increased expression, transcription activation of involved genes, and activity of efflux drug transporters represent a major mechanism for developing chemoresistance, mostly under the control of epigenetic processes like DNA methylation, histone acetylation, and ncRNA interaction (**Figure 4**) (142, 144, 148, 149). The involvement of lncRNAs in drug metabolism and efflux phases has been investigated to elucidate the DR mechanisms of cancer (141).

Some studies have demonstrated the involvement of lncRNAs in the metabolism and disposition of anti-cancer drugs, influencing directly the development of DR (142, 148). In HNSCC, DR has become an increasingly concerning challenge for the scientific community and clinicians (16, 31, 135). Along with many others, the long intergenic non-coding RNA 00958 (LINC00958) has been studied as apoptosis- and autophagy-related lncRNA in HNSCC as part of prognostic signature, establishing a worse overall survival rate of patients when the signature is present (140, 150). Moreover, Jian et al. concluded that LINC00958 downregulates miR-4306 levels to activate the pyroptosis pathway mediated by AIM2 and promotes cancer cell survival in OSCC (96). Another study suggests that LINC00958 interplay with c-Myc as a feedback loop facilitating HNSCC development and resistance to chemo- and radiotherapy, and its upregulation is associated with poor tumor differentiation, advanced tumor stage, and shorter overall survival of patients (31) Another lncRNA related to DR is the plasmacytoma variant translocation 1 (PVT1), identified as upregulated in cisplatin-resistant cancer cell lines and tissue samples (108) and as a promoter of decreased sensitivity to cetuximab (135). **Table 2** summarizes the most important dysregulated lncRNAs that influence DR on HNSCC.

**Table 2:** LncRNAs and their influence on HNSCC drug resistance.

LNCRNA	INFLUENCE	REFERENCE
**OSCC**		
**UCA1**	Accelerates proliferation, increases CDDP chemoresistance, and restrains apoptosis.	(97)
**HOXA11-AS**	Promotes proliferation in CDDP-sensitive cells and inhibits CDDP-induced cytotoxicity through the HOXA11-AS/miR214-3p/PIM1 axis.	(98)
**XIST**	Upregulation of XIST promotes cell proliferation, enhances CDDP resistance, and inhibits apoptosis.	(99)
**MALAT1**	Induces EMT and CDDP resistance *via* activation of PI3K/AKT/m-TOR signaling pathway and the upregulation of P-gp.	(100)
**ANRIL**	CAF-secreted midkine enhances tumor cell resistance to cisplatin by inducing ANRIL expression and increasing anti-apoptotic protein Bcl-2 expression.	(101)
**OIP5-AS1**	Its knockdown enhances DDP sensitivity *in vivo*. Improves DDP resistance through the upregulation of TRIM14 mediated by miR-27b-3p.	(102)
**KCNQ1OT1**	Promotes DDP resistance of tongue cancer by sponging miR-124-30 to regulate TRIM14 expression. Facilitates tumor growth and chemo-resistance by acting as a competing endogenous RNA (ceRNA) to modulate the expression of miR-211-5p.	(28) (103)
**SNHG26**	Its expression is positively correlated with proliferation, EMT, migration, invasion, and cisplatin resistance by binding directly to PGK1 protein, inhibiting its ubiquitination and activating the Akt/mTOR signaling pathway.	(104)
**CYTOR**	Acts as a ceRNA to inhibit miR-1252-5p and miR-3148 upregulating LPP expression. CYTOR/LPP axis is essential for FOXD1-induced EMT and chemoresistance.	(15)
**LHFLP3-AS1**	It is upregulated in cisplatin-resistant tumors promoting proliferation, migration, and invasion.	(105)
**CEBPA-DT**	Regulates cisplatin chemosensitivity through CEBPA-BCL12-mediated cell apoptosis.	(106)
**MPRL**	High expression of MPRL and pre-miR-483 and low expression of miR-143-5p are associated with neoadjuvant chemosensitivity and better prognosis.	(107)
**PVT1**	Its upregulation in cisplatin-resistant tissues and cell lines is strongly correlated with worse overall survival acting as a ceRNA on miR-194-5p.	(108)
**HEIH**	Exosomal HEIH acts as a ceRNA for miR-3619-5p to upregulate HDGF, promoting DDP resistance.	(109)
**CILA1**	High CILA1 expression levels and low levels of phosphorylated beta-catenin are associated with cisplatin resistance and advanced disease stage.	(110)
**APCDD1L-AS1**	Its expression is related to worse prognosis and confers resistance to 5-FU *via* miR-1224-5p/NSD2 axis.	(111)
**TUG1**	Its upregulation promotes cisplatin resistance by mediating miR-133b and CXCR4.	(112)
**LINC00963**	Its suppression reduces the activity of ALDH1, percentage of self-renewal, chemoresistance and expression of multidrug-resistance transporter ABCB5.	(17)
**NPC**		
**HOXA11-AS1**	Enhances DDP resistance *via* the miR-98/PBX3 axis. Its silencing inhibits the c-Met/AKT/mTOR pathway by specifically upregulating miR-454.3p, promoting cell apoptosis and enhancing the sensitivity of cisplatin-resistant cells.	(116) (117)
**KCNQ1OT1**	Enhances DDP resistance, proliferation, migration, and invasion *via* the miR-454/USP47 axis.	(118)
**MIAT**	Upregulates HMB1 expression, contributing to cisplatin resistance and poor clinical outcome *via* the MIAT/HMGB1/IL6 axis.	(121)
**NEAT1**	NEAT1/let-7a-5p axis regulates the cisplatin resistance by targeting Rsf-1 and modulating the Ras-MAPK signaling pathway. NEAT1 increases in tissues and manages to facilitate SAHA tolerance by modulating the miR-129/Bcl-2 axis.	(122) (126)
**n375709**	Its inhibition increases the paclitaxel sensitivity.	(125)
**MAGI2-AS3**	MAG2-AS1/mR-218-5p/GDPD5/SEC61A1 axis drives cell proliferation, migration, and EMT, and conferred cisplatin resistance.	(124)
**LINC00346**	LINC00346 regulates the cisplatin resistance by inhibiting miR-342-5p.	(123)
**TINCR**	Acts as a crucial driver of progression and chemoresistance, and highlights the INCR-ACLY-PADI1-MAPK-MMP2/9 axis.	(119)
**CCAT1**	Its upregulation enhances paclitaxel resistance *via* miR-181a/CPEB2 axis.	(127)
**MRVI1-AS1**	MRVI1-AS1/ATF3 signaling pathway increases paclitaxel chemosensitivity by modulating the Hippo-TAZ.	(128)
**DLEU1**	Acts as an oncogene to promote DDP resistance and BIRC6 expression through interacting with miR-381-3p.	(129)
**LSCC**		
**H19**	Exerts inhibiting effect on autophagy and drug resistance by downregulating HMGB1 through targeting miR-107.	(131)
**MALAT1**	Its over-expression enhances chemoresistance and demonstrates poorer 5-year overall survival.	(132)
**FOXD2-AS1**	FOXD2-AS1 acts as a scaffold for STAT3 and PRMT5, promoting STAT3 transcriptional activity, essential to maintain cancer stemness and promote chemotherapeutic resistance.	(18)
**FGD5-AS1**	Its overexpression increases cisplatin-resistance by modulating miR-497-5p/SEPT2 axis.	(133)
**HOXA11-AS1**	Enhances CDDP resistance of LSCC *via* miR-518a/SPATS2L axis. Its knockdown inhibits viability, migration, and invasion, but promoted apoptosis.	(134)
**LINC-PINT**	Targets miR-425-5p which also targeted PTCH1, affecting the Hedgehog pathway, thus increasing cancer stemness and chemoresistance to cisplatin.	(19)
**AFAP1-AS1**	Increases RBPJ expression by negatively regulating miR-320a and RBPJ overexpression rescues stemness and chemoresistance inhibited by AFAP1-AS1 silencing.	(120)
**BANCR**	Its downregulation reverses cisplatin resistance.	(30)
**HNSCC**		
**PVT1**	Decreases the sensitivity of HNSCC cells to cetuximab by enhancing methylation-mediated inhibition of miR-124-3p.	(135)
**LINC00461**	Downregulates expression of miR-195 to subsequently upregulate expression of HOXA10, promoting EMT and enhancing chemoresistance.	(16)
**Lnc-POP1-1**	lnc-POP1-1 promotes DNA repair through interaction with MCM5 and deceleration of its degradation. VN1R5 promotes cisplatin resistance in a lnc-POP1-1-dependent manner.	(32)
**LINC00958**	LINC00958 interplays with c-Myc as a feedback loop facilitating development and resistance to chemo- and radiotherapy.	(31)

The homeobox A11 antisense lncRNA (HOXA11-AS) has also been related to chemoresistance. In this regard, OSCC tumor tissues and cell lines were analyzed, concluding that the upregulation of HOXA11-AS promoted proliferation in CDDP-sensitive cells and inhibited CDDP-induced cytotoxicity by intervention in the miR214-3p/PIM1 axis (98). Other studies demonstrated that the knockdown of HOXA11-AS enhances CDDP resistance *via* the miR-98/PBX3 axis (116) and can inhibit the c-Met/AKT/mTOR pathway by specifically upregulating miR-454-3p, thus promoting cell apoptosis and enhancing the sensitivity of cisplatin-resistant NPC cells to cisplatin (117). Conversely, Shen et al. analyzed LSCC tissues and cell lines, showing that HOXA11-AS1 knockdown inhibits the viability, migration, and invasion but promotes the apoptosis of cells (134).

Another lncRNA actively involved in DR in HNSCC is the KCNQ1 overlapping transcript 1 (KCNQ1OT1). It has been established that its upregulation facilitates tumor growth and chemoresistance in tongue SCC by sponging miR-124-3p (28) and by acting as a ceRNA to modulate the expression of miR-211-5p (103). Moreover, Yuan et al. showed that KCNQ1OT1 knockdown promotes chemosensitivity in DDP-resistant NPC cells by significantly decreasing cell proliferation, migration, and invasion *via* the miR-454/USP47 axis (118). NEAT1 (nuclear paraspeckle assembly transcript 1) has also been associated with HNSCC chemoresistance, especially in NPC where its depletion repressed the cisplatin resistance of NPC cells and phenocopied the effect of miR-129 overexpression, which also enhanced apoptosis by the histone deacetylase inhibitor SAHA (122, 126).

The metastasis-associated lung adenocarcinoma transcript 1 (MALAT1) has been identified as a prognostic factor in patients with lung cancer (151), and its overexpression is related to poor clinical outcome, chemoresistance, and progression in many cancer types, including kidney (152, 153), pancreatic (154), prostate (155, 156), esophageal (157, 158), breast (159, 160), gastric (161), ovarian (162), and colorectal (163, 164). In both oral and laryngeal SCC, the over-expression of MALAT1 contributes to the enhanced chemoresistance and metastatic power of several cell lines (100, 132).

In the case of H19, it has been demonstrated that it is upregulated in LSCC, exerting an inhibiting effect at the autophagy level and DR by downregulating HMGB1 by targeting miR-107 (131). A similar effect has been observed in many other cancer types, with anti-apoptotic, pro-proliferative, and pro-migratory functions, along with the induction of EMT, activation of oncogenic signaling pathways, and changes in the tumor microenvironment, contributing to anti-cancer DR (165). Another well-studied lncRNA that promotes the proliferation, migration, and chemoresistance of several cancer types is the myocardial infarction-associated transcript (MIAT) (166–169). According to Zhu et al., an elevated MIAT level upregulates HMB1 expression, contributing to cisplatin resistance and poor clinical outcomes (121).

An increasing number of other lncRNAs have been proposed as promoters of DR in HNSCC. For instance, the urothelial cancer-associated lncRNA 1 (UCA1) plays an important role in the tumorigenesis, progression, and diagnosis of many cancers, mainly bladder cancer (170, 171). In oral cancer tissues and cell lines, UCA1 accelerates proliferation, increases CDDP chemoresistance and restrains apoptosis partly by modulating SF1 by sponging miR-184 (97). In another study, the results showed that CAF-secreted midkine enhanced OSCC resistance to cisplatin by inducing ANRIL expression and increasing the anti-apoptotic protein Bcl-2 expression (101). Chen et al. studied 146 paraffin-embedded OSCC specimens along with OSCC cell lines CAL-27 and SCC4 and found that the cytoskeleton regulator RNA (CYTOR) acts as a ceRNA to inhibit miR-1252-5p and miR-3148, upregulating the lipoma-preferred partner (LPP) protein and therefore proving essential for FOXD1-induced EMT and chemoresistance (15). Another forkhead box (FOX) reported as important for laryngeal SCC DR is FOXD2-AS1, which acts as a scaffold for STAT3 and PRMT5, promoting STAT3 transcriptional activity, maintaining cancer stemness, and promoting chemotherapeutic resistance (18).

### LncRNAs in autophagy

The intracellular degradation systems encompass two major protein pathways that are directly involved in the maintenance of metabolic homeostasis; one of them is the ubiquitin–proteasome pathway, responsible for degrading short-lived and damaged proteins; and the other is the lysosome-autophagy system, whose target is long-lived macromolecular complexes and organelles (172). Autophagy is a highly conserved and successive cellular process in which damaged organelles, intracellular microbes, and pathogenic long-lived proteins are degraded, recycling amino acids, nucleotides, and fatty acids to maintain cellular homeostasis (173–175). Thus, autophagy is closely related to the occurrence of a wide variety of human diseases (176).

Autophagy-related genes (ATGs) are responsible for autophagy occurring under micro-environmental stress such as hypoxia, heat, nutrient deficiency, and accumulation of reactive oxygen species (175, 176). The main successive autophagy stages are the initiation of phagophore assembly, autophagosomal formation, and lysosomal fusion (177, 178). Two highly conserved serine–threonine kinases, the mammalian target of rapamycin (mTOR) and the mammalian homologs of yeast ATG1-Unc-51-like kinases 1 (ULK1), regulate cell growth and survival and are the central modulators of autophagy, responding to intra- and extra-cellular changes by forming autophagosomes (179, 180). mTOR is activated under favorable conditions, inhibiting autophagy and protein degradation, whereas it is inactivated in hostile environments related to poor nutritional conditions (172).

Recent studies have demonstrated an important role for autophagy in tumorigenesis and the progression of cancer (181). A dual function has been proposed since autophagy can stabilize the genome and prevent the formation of tumor cells, while once the tumor cells have been formed, autophagy plays a pivotal role in tumor initiation, progression, and resistance to chemotherapy (172, 177). LncRNAs are involved in autophagy, modulating the expression of ATG genes by acting as ceRNAs for miRNAs (36, 180). Recently, several autophagy-related lncRNAs have been proposed as biomarkers and signatures for diagnostic and prognostic purposes for many cancer types, including breast (182, 183), bladder (184, 185), pancreatic (186), colorectal (187, 188), and lung (189, 190). In most cases, the overall survival of patients in high-risk groups is significantly lower based on the presence of each proposed signature.

Many studies have established prognostic signatures in HNSCC. For instance, in a recent study, 910 autophagy-related (AR) lncRNAs from mRNA sequences and clinical data of HNSCC patients and controls from The Cancer Genome Atlas (TCGA) were analyzed. The principal component analysis distinguished two categories based on the nine lncRNA prognostic signatures, resulting in a significantly worse overall survival rate in the high-risk group (150). Another study consulted the TCGA database to obtain 155 HNSCC samples (mainly laryngeal, nasopharyngeal, tonsil, and lip cancer) and the RNA profile indicated that ATG12, BECN1, and MAP1LC3B have prognostic value, and their related pathways may be involved in regulating HNSCC prognosis (191). Guo et al. included 17 prognostic-related autophagy- and ferroptosis-related lncRNAs as the main components of a ceRNA network that regulates differentially expressed genes mainly enriched in immune-related pathways (12). Similarly, gene set enrichment analysis (GSEA) and gene ontology (GO) functional annotation proved that autophagy-related pathways are mainly enriched when 13 autophagy-related lncRNAs are present in HNSCC patients (140). Regarding oral and oropharyngeal SCC, the signature-based on nine autophagy-related lncRNAs acted as an independent prognostic indicator, showing a significantly lower overall survival in high-risk groups (13). The autophagy-related (AR) signatures of lncRNAs proposed as biomarkers in HNSCC are summarized in **Table 3**.

**Table 3:** Autophagy-related (AR) signatures of lncRNAs proposed as biomarkers in HNSCC.

AR LNCRNAS	INFLUENCE	REFERENCE
**PTCSC2**, **AC099850.3**, **LINC01963**, **RTCA-AS1**, **AP002884.1**, **UBAC2-AS1**, **AL512274.1**, **MIR600HG**, **AL354733.3**	The overall survival of the high-risk group was significantly lower than that of the low-risk group. The signature-based on autophagy/related lncRNAs potentially acts as an independent prognostic indicator for patients with OSCC/OPSCC.	(13)
**ATG12**, **BACN1**, **MAP1LC3B**	All three autophagy-related lncRNAs have prognostic value with respect to HNSCC, and their related pathways may be involved in regulating HNSCC prognosis.	(191)
**MIR4435-2HG**, **PCED1B-AS1**, **AL512274-1**, **MYOSLID**, **LINC01871**, **LINC02541**, **AC012236-1**, **C5orf66-AS1**, **AC004687-1**, **AL354836.1**, **LINC02454**, **AC024075.2**, **LINC00460**, **AATBC**, **ITGB2-AS1**, **MIR9-3HG**, **AF131215.5**	Differentially expressed genes (DEGs) between high- and low-risk groups were mainly enriched in immune-related pathways and regulated by a PAF-lncRNA-directed ceRNA network.	(12)
**AC008115.3**, **AL139158.2**, **AC136475.2**, **AL160006.1**, **AL357033.4**, **AC007991.2**, **AC104083.1**, **AL139287.1**, **AL450992.2**, **LINC00958**, **AC103702.2**, **PSMA3-AS1**, **UBAC2-AS1**	Overall survival in the high-risk group was shorter than the low-risk group. Gene set enrichment analysis (GSEA) and gene ontology (GO) functional annotation proved that autophagy-related pathways are mainly enriched in the high-risk group.	(140)

Quantitative reverse transcription PCR (RT-qPCR) was performed to analyze the cancer susceptibility candidate 9 (CASC9) expression in OSCC tissues and cell lines, demonstrating that CASC9 promotes progression by enhancing cell proliferation and suppressing autophagy-mediated cell apoptosis *via* the AKT/mTOR pathway (92). Another study by Chen et al. showed that when the gastric cancer-associated transcript 1 (GACAT1) was inhibited in OSCC samples, it promoted apoptosis and autophagy, mainly related to the targeting of miRNA-149 (93). Long intergenic non-coding RNA 01207 (LINC01207) and LINC00958 were also upregulated in OSCC tissues and cells. LINC01207 upregulates LDHA expression to promote cell proliferation and migration and inhibits apoptosis and autophagy by acting as a ceRNA that sponges miR-1301-3p (94), whereas LINC00958 downregulates miR-4306 levels to activate a pyroptosis pathway mediated by AIM2 and promotes cancer cell survival (96).

A broadly studied oncogenic trans-acting lncRNA is the HOX transcript antisense RNA (HOTAIR), which is found overexpressed in a wide variety of cancers and is mainly associated with metastasis and poor prognosis (192). In OSCC cells, HOTAIR silencing inhibited autophagy with the downregulated expression of MAP1LC3B, Beclin1, ATG3, and ATG7; proliferation, migration, and invasion of OSCC cells were suppressed, along with an enhanced apoptosis rate and an improvement in sensitivity to cisplatin (95).

As previously addressed, MEG3 is considered a tumor-suppressor lncRNA. Lin et al. concluded that MEG3 promotes autophagy and apoptosis of NPC cells by enhancing PTEN expression by binding to miR-21 (113). Another important tumor-suppressor lncRNA involved in autophagy activation is the growth arrest-specific 5 RNA (GAS5). In LSCC, GAS5 inhibited the viability of AMC-HN-8 cells and induced apoptosis, acting as a tumor suppressor by regulating the miR-26a-5o/ULK2 axis (130). Conversely, CASC19 suppressed cellular autophagy by inhibiting the AMPK/mTOR pathway, contributing to the radioresistance of NPC by regulating autophagy (114).

### LncRNAs in EMT

Epithelial-to-mesenchymal transition (EMT) is described as a process where epithelial cells are transformed into mesenchymal stem cells and plays an important role in both development and tumorigenesis (177, 193). Moreover, EMT has been broadly related to tumor proliferation, metastasis, and DR (194, 195). However, this transition is reversible since tumor cells will go through the opposite process of mesenchymal-to-epithelial transition (MET) once they have reached a suitable place where they can metastasize, re-expressing epithelial characteristics (196). A wide variety of signaling pathways can be involved in EMT, including the transforming growth factor-beta (TGF-beta) pathway (196, 197), the Wnt/beta-catenin pathway (198, 199), the Notch signaling pathway (200), the Hedgehog pathway (201), and the signal transducer and activator of transcription 3 (STAT3) pathway (202), among others. These signaling molecules can subsequently activate different EMT transcription factors like Snail, basic helix-loop-helix (TWIST), and zing-finger E-box-binding homeobox (ZEB) to repress epithelial markers and activate the EMT program (6, 177, 196).

Recently, several lncRNAs have been linked to EMT since they play fundamental roles in the above-mentioned signaling cascades, epigenetics, and transcription factors (203–205). The lncRNA MALAT1 induces EMT and CDDP resistance in OSCC cells *via* the activation of the PI3K/AKT/mTOR signaling pathway in cell lines CAL-27 and SCC-9 (100). LncRNA KCNQ1OT1 has also been related to EMT in tongue cancer tissues and cells, promoting survival rate proliferation, migration, and invasion (28). Similarly, quantitative PCR performed in pituitary adenoma samples found the same lncRNA to be upregulated, and its knockdown inhibited cell stemness, angiogenesis, and EMT (206).

A novel lncRNA named chemotherapy-induced lncRNA 1 (CILA1) was upregulated in cisplatin-resistant tongue SCC cells, displaying EMT features, promoting invasiveness and chemo-resistance, mainly activating the Wnt/beta-catenin pathway (110). Another lncRNA related to EMT is the recently discovered small nucleolar RNA host gene 26 (SNHG26), first described by Tong et al. as part of a prognosis signature along with the other 13 lncRNAs in bladder cancer (207). Similarly, a four-lncRNA signature that included SNHG26 was associated with immune infiltration and prognosis in colon cancer; the signature-divided colon cancer patients of TCGA into high- and low-risk groups with significantly different outcomes (208). In TSCC, SNHG26 expression was positively correlated with proliferation, EMT, migration, invasion, and cisplatin resistance by activating the AKT/mTOR signaling pathway (104).

Another lncRNA recently linked to EMT in cancer is the long intergenic non-protein coding RNA 461 (LINC00461), highly expressed in non-small cell lung cancer and directly involved in cell proliferation, migration, and EMT by targeting the miR-4478/E2F1 axis (209). Similarly, Wu et al. showed that the upregulation of LINC00461, LINC00402, and SFTA1P had suppressive effects on the homologous pleckstrin-homology (PH)-domain leucine-rich-repeat protein phosphatases (PHLPP2), reported previously as a tumor suppressor in colon cancer (210). In HNSCC, LINC00461 was highly expressed in 52 tissues analyzed, and it was found that LINC00461 downregulates the expression of miR-195 to subsequently upregulates the expression of HOXA10, promoting EMT and enhancing chemoresistance in HSNCC (16).

The membrane-associated guanylate kinase 2 antisense RNA 3 (MAGI2-AS3) was recently identified in NPC, driving cell proliferation, migration, and EMT through the miR-218-5p/GDPD5/SEC61A1 axis, conferring cisplatin resistance in cell lines (124). The same lncRNA expression was detected by quantitative real-time PCR in pancreatic cancer cells, and its upregulation promoted EMT through the regulation of miR-490-5p (211). Moreover, MAGI2-AS1 was identified to be EMT-related and highly co-expressed with ZEB1/2 in both gastric tissues and normal stomach tissue (212). Conversely, MAGI2.AS3 overexpression inhibited bladder cancer progression by regulating MAGI2/PTEN/EMT in 80 bladder cancer tissues (213).

Although several studies have encompassed the involvement of lncRNAs as regulators of EMT and, consequently, DR in HNSCC, more studies must complement the information available at present time.

### LncRNAs in stemness

The stemness of cancer cells is an important cellular feature that grants tumor heterogeneity, enhanced growth capacity, DR, and augmented metastatic ability through the CSC properties of self-renewal, quiescent state, high cell turnover, increased expression of drug transporters, and other resistance genes (177, 214, 215). Evidence suggests that CSCs retain properties that make them highly resistant to currently available chemotherapy drugs since CSCs remain in an inactive state of the cell cycle and most of these treatments attack cells with a high proliferative rate (216, 217). Additional properties like rapid DNA repair (218), tumor microenvironment (219, 220), and extracellular matrix contribute to maintaining cancer stemness and chemoresistance (221, 222).

As previously addressed, even though lncRNAs have no protein-coding capacity, they are emerging as master regulators of gene transcription and act as proto-oncogenes or tumor suppressors (223, 224). LncRNAs like LINC00617 (225), lncSOX2OT (226), and HOTAIR (227, 228) play an important role in CSC regulation of various types of cancer through several mechanisms and signaling pathways. Furthermore, in colorectal cancer, the lncTCF7 interacts with subunits of the SWI/SNF chromatin remodeling complex, regulating the transcription of the TCF7 gene and activating the Wnt cascade, involved in stem cell self-renewal (229). H19 is another lncRNA overexpressed in many cancers and confer stem-like properties in correlation with stem cell markers like SOX2, OCT4, NOTCH1, c-Myc, and ABCG2 (230–232).

Little research has been done concerning the role of lncRNAs in DR associated with cancer cell stemness. Lee et al. studied OSCC tumor tissues and cell lines and concluded that the downregulation of the long intergenic non-coding RNA 963 (LINC00963) inhibited CSC hallmarks, such as migration, invasion, and colony formation capacity. Moreover, the suppression of LINC00963 reduced the activity of ALDH1, the percentage of self-renewal, chemoresistance, and the expression of multidrug-resistance transporter ABCB5 (17). Another long intergenic non-coding RNA recently involved in chemoresistance driven by cancer cell stemness is the p53-induced non-coding transcript (LINC-PINT). Interestingly, the tumor suppressor PTCSC3 was studied along with LINC-PINT in gastric cancer tissues, inhibiting tumor growth and stemness when both were over-expressed (233). The same lncRNA was observed in 24 LSCC samples and cells, targeting miR-425-5p and subsequently targeting PTCH1, affecting the Hedgehog pathway and its downregulation was associated with increased cancer stemness and chemoresistance to cisplatin (19).

LncRNA FOXD2-AS1 has been related to different forms of malignancy and CSCs, mainly involving gliomas (234). In laryngeal SCC chemotherapy-resistant patients, FOXD2-AS1 showed increased expression and acted as a scaffold for STAT2 and PRMT5, both essential to maintain cancer stemness and promote chemotherapy resistance (18). Oncogenic actin filament-associated protein 1-antisense RNA 1 (AFAP1-AS1) is a recently discovered lncRNA related to cancer stemness (235). The first documented association between AFAP1-AS1 high expression, stemness, and DR was found in LSCC specimens, increasing RBPJ expression by negatively regulating miR-320a; subsequently, RBPJ overexpression rescued stemness and chemoresistance inhibited by AFAP1-AS1 silencing (120). Another study suggested that AFAP1-AS1 functions as an endogenous RNA by competitively binding to miR-384 to regulate ACVR1, thus conferring inhibitory effects on pancreatic cell stemness and tumorigenicity (236).

## Clinical relevance of lncRNAs in drug resistance mechanisms in HNSCC

The clinical importance of establishing a correlation between the increasing number of newly discovered lncRNAs and the various mechanisms of DR lies in the implications that they have on the prognosis, molecular staging, and treatment possibilities of many tumors. In this regard, CASC9 was strongly associated with tumor size, clinical stage, regional lymph node metastasis, and overall survival time in OSCC patients (92). Similarly, the upregulation of PVT1 was strongly correlated with the worse overall survival of 83 OSCC patients (108). Lin et al. proposed CILA1 as a biomarker in TSCC, correlating its expression levels with cisplatin resistance and advanced disease stage (110). Moreover, SNHG26 expression was also associated with the occurrence, progression, and poor prognosis of TSCC (104).

It is important to remember that DR can be categorized as intrinsic and acquired resistance, the first being defined as the lack of tumor regression following treatment (which is the result of mechanisms that existed before therapy). Meanwhile, acquired resistance denotes the elimination of an observed response after an initial clinical benefit following treatment (237). Because stemness of cancer cells is an intrinsic mechanism of DR, its clinical relevance stands out given that lncRNAs such as LINC00963, LINC-PINT, FOXD2-AS1, and AFAP1-AS1 have been found overexpressed, conferring the stemness state to cells. Nevertheless, to date, there is no evidence of a relationship between these specific lncRNAs and a particular drug.

As prognostic signatures, several studies have linked lncRNAs with the prognosis of HNSCC, particularly autophagy- and ferroptosis-related lncRNAs (12, 13, 140, 150, 191). In all cases, the signatures proposed exhibited prognostic value concerning HNSCC (**Table 3**), and their related pathways may be involved in regulating HNSCC prognosis.

## Future directions for lncRNA research

The role of lncRNAs in many diseases has become a widely investigated field, especially in cancer research. Given the increasing evidence of the involvement of lncRNAs in several drug resistance mechanisms, research should be directed toward new horizons to elucidate the molecular pathways by which lncRNAs interact to drive the resistance of certain cell lines (51). This understanding would help in the improvement of the diagnosis and treatment strategies of HNSCC (3, 238). A potential line of research could involve the upstream regulatory mechanisms of lncRNAs since previous evidence suggests regulation by histone status, DNA methylation patterns (107), transcription factors (239), and post-transcriptional regulation (240, 241).

With the development of high-throughput sequencing technology, the library of lncRNAs has notably increased. However, most of the mechanisms of influence on DR through phenomena such as EMT (15), autophagy (131), and stemness (18) have not been fully understood, and some even remain unexplored. A better comprehension of the regulatory networks between lncRNAs, genetic, and epigenetic alterations could give rise to therapeutic strategies that promote improvements in dealing with DR mechanisms (107). Also, the elucidation of all regulatory networks could lead to the formulation of clinical trials targeting specific lncRNAs.

To achieve the clinical application of lncRNAs, molecular techniques such as microarrays, RNA-seq, and qRT–PCR (242) have been used to quantify their expression, but still numerous limitations that need to be overcome. For instance, technical procedures such as ensuring stability, sample preparation, lncRNA extraction, and detection must be standardized. Besides, the sensitivity and specificity of lncRNAs must be ensured. Thus, until all the technical difficulties have been overcome, the detection of circulating lncRNAs would be applied in regular clinical practice (243).

## Conclusion

In recent years, the pivotal role of lncRNAs in DR has begun to gain importance in the mechanisms that harbor and promote chemoresistance in HNSCC. As the involvement of more lncRNAs is elucidated, an improvement in diagnostic and prognostic tools could promote an advance in targeted and specific therapies in precision oncology.

## Author contributions

JP-F, MB, and RR-P conceived and designed the content of this review. JP-F, MB, CV-M, US-B, DM-C, AA-R, BP-A, AL-P, CL-C, JL-G, JG-P, ME-M, JC-Q, and AB-G wrote the paper. All authors contributed to the final version of the paper and approved the submitted version.

## Conflict of interest

The authors declare that the research was conducted in the absence of any commercial or financial relationships that could be construed as a potential conflict of interest.

## Publisher’s note

All claims expressed in this article are solely those of the authors and do not necessarily represent those of their affiliated organizations, or those of the publisher, the editors and the reviewers. Any product that may be evaluated in this article, or claim that may be made by its manufacturer, is not guaranteed or endorsed by the publisher.
